# Draft Genome Sequence of a *Salmonella enterica* subsp. *enterica* Serovar Gallinarum bv. Gallinarum Isolate Associated with Fowl Typhoid Outbreaks in Brazil

**DOI:** 10.1128/genomeA.00019-16

**Published:** 2016-02-25

**Authors:** Silvia De Carli, Tiago Gräf, Fabiana Q. Mayer, Samuel Cibulski, Fernanda K. M. Lehmann, André S. K. Fonseca, Nilo Ikuta, Vagner R. Lunge

**Affiliations:** a Laboratório de Diagnóstico Molecular, Universidade Luterana do Brasil (ULBRA), Canoas, Rio Grande do Sul, Brazil; b Laboratório de Biologia Molecular, Instituto de Pesquisas Veterinárias Desidério Finamor (IPVDF), Fundação Estadual de Pesquisa Agropecuária, Eldorado do Sul, Rio Grande do Sul, Brazil; c Simbios Biotecnologia, Cachoeirinha, Rio Grande do Sul, Brazil

## Abstract

*Salmonella enterica* subsp. *enterica* serovar Gallinarum bv. Gallinarum strains are bird pathogens causing fowl typhoid (FT). Isolate BR_RS12 was obtained from a poultry flock with FT in 2014. The sequencing of this genome will enable to track the origin of the recent outbreaks in Brazil.

## GENOME ANNOUNCEMENT

Fowl typhoid (FT) is an avian disease caused by *Salmonella enterica* subsp. *enterica* serovar Gallinarum bv. Gallinarum. FT was reported in adult birds presenting several clinical signs in poultry flocks worldwide (1, 2). Epidemiological surveillance and biosecurity efforts eradicated this pathogen in the main poultry-producing countries, partially due to the use of the live vaccine SG9R (3). However, 82 FT outbreaks were reported in South America in the last few years (2). Two possibilities were raised to explain this high incidence: the latency of *S*. Gallinarum in backyard chickens and conversion of the live vaccine to a virulent form (4, 5). The current study reports the genome sequence of an *S*. Gallinarum isolate from a recent outbreak in South Brazil to further compare the virulence profiles and genetic evolution of *S*. Gallinarum strains over the last few years.

Fifteen typical *S*. Gallinarum isolates were obtained from birds with FT in different poultry flocks in South Brazil in 2014. All samples were first enriched with buffered peptone water (BPW) at 36°C for 24 h, followed by selective enrichment in Rappaport-Vassiliadis and tetrathionate broths at 43°C for another 24 h. Afterward, they were plated on MacConkey agar, and typical colonies were observed after 24 h at 36°C. The isolates were serotyped according to the Kauffman-White scheme (6) and biochemically tested for ornithine and lysine decarboxylation, hydrogen sulfide production, urea decomposition, and the use of sucrose, lactose, maltose, and citrate (1, 7, 8).

The isolate *Salmonella* BR_RS12 was selected to genome sequencing analysis. DNA was extracted using PureLink genomic DNA minikit (Thermo Fisher Scientific, Waltham, MA). A library was prepared with the Nextera kit (Illumina, San Diego, CA, USA), and sequencing was performed with MiSeq platform (Illumina). Quality analysis was conducted using the FastQC software (version 0.11.4 [http://www.bioinformatics.babraham.ac.uk/projects/fastqc]), and sequence positions with a quality score of <30 were trimmed out using Sickle (version 1.33; GitHub). Best kmer length was estimated by using KmerGenie (9), and *de novo* assembly of the genome was performed with SPAdes (10). The quality of the assembled contigs was assessed with QUAST (11), and the sequence was annotated with RAST (12), using the AM933173 reference sequence (13).

A total of 844,742 paired-end reads were obtained, and fragments ranged from 35 to 301 bp, with 52% G+C content. *De novo* assembly generated 41 contigs, with the biggest being 806,884 bp long, resulting in ~180× coverage. Using 34 contigs >500 bp, a complete genome of 4,702,900 bp was mounted, with an *N*_50_ of 400,827 bp. The annotation process identified 4,824 coding sequences (CDSs), 69 tRNAs, and 14 rRNAs. Further, two prophages (49,000 and 37,800 bp) were identified with the software Phast (14), and the plasmid pSG (88,450 bp) was detected in a BLAST search using the nonassembled contigs as queries (http://blast.ncbi.nlm.nih.gov).

The current announced genome is the first *S*. Gallinarum field strain sequenced in this century in South America. This information will be useful to track down the source of the frequent FT outbreaks in Brazil.

### Nucleotide sequence accession numbers.

The draft genome was deposited in NCBI under BioProject accession no. PRJNA302466 and BioSample accession no. SAMN04274130. This description is of the first version, LNON00000000.
